# Vegetable Tannins Used in the Manufacture of Historic Leathers

**DOI:** 10.3390/molecules23051081

**Published:** 2018-05-03

**Authors:** Lina Falcão, Maria Eduarda M. Araújo

**Affiliations:** 1 Artistic Studies Research Centre, Faculty of Fine Arts, University of Lisbon, Largo da Academia Nacional de Belas-Artes, 1249-058 Lisboa, Portugal; linafalcao@gmail.com; 2 Centre of Chemistry and Biochemistry, Department of Chemistry and Biochemistry, Faculty of Sciences, University of Lisbon, Campo Grande, Edifício C-8, 1749-016 Lisboa, Portugal

**Keywords:** tannins, vegetable tanning, European historic leathers, colorimetric tests, spectroscopy, UV-Vis, FTIR

## Abstract

In this review, a brief description of how animal skins were transformed in leathers in Europe using different vegetable tannins will be presented. Special attention will be dedicated to the description of the type of tannins and the characteristics of the most important type of historic leathers thus obtained. The text will also focus on the description of the techniques used in the identification of these tannins in historic objects: colorimetric tests and spectroscopic analysis.

## 1. Introduction

Natural tannins, stricto sensu, are polyphenolic compounds of vegetal origin with the property to precipitate proteins. It is assumed that the oldest application of this tannins chemical property in technology is the stabilization of animal skins protein against putrefaction [1].

According to some authors [2,3,4,5], it may have been by the end of Neolithic period and in the eastern Mediterranean region that man began to use, incipiently, plant materials, such as leaves, twigs, fruits, barks or roots, to prevent animal skins degradation, transforming them into a more durable and useful material. Empirical development of this process during Classical Antiquity resulted in one of the most important animal skin transformation technique: vegetable tanning. This millenary technology, which is still in use, is one of the oldest processes used to produce leather. It was introduced in the north western regions of Europe after the Roman conquest [6], reaching thereafter a great importance in leather production which lasted until the end of the 19th century, when mineral tanning based on chromium (III) salts was developed and implemented in tanneries. Mineral tanning is nowadays the most used tanning process. In the early of the 20th century synthetic tannins, based on different organic molecules, were introduced in leather industry with the purpose of aiding vegetable tanning [1,7].

Succinctly, the vegetable tanning method can be described as the treatment of animal skin—previously washed, limed, dehaired, fleshed, delimed-, with crushed vegetable materials, or liquors/macerations (oozes), infusions, or decoctions, or extracts prepared with those materials. The result is the transformation of animal skin into what is termed by leather: a stable and non-putrescible material, resistant to deterioration promoted both by microorganisms and heat, when wetted. This is due to the chemical bonds established between collagen, the main constitutive protein of skin, and the tannins present in the vegetable materials. Collagen stabilization occurs when 15% to 40% of tannins, per dry weight of skins, are absorbed and incorporated into the collagen fibres matrix, depending of the type of leather produced [1,8,9,10].

The origin of the term ‘tannin’ it is also close related to the vegetable tanning process and came into use in France at the end of the 18th century by the French chemist Armand Séguin (1767–1835), who had previously worked with Antoine Lavoisier. Séguin firstly used the word *tannin* to define the organic substance present in the aqueous extracts of plants responsible for the transformation of skin into leather. The word derives from the French word *tan*, which corresponds to the ground oak bark, an important widespread and extensively used European vegetable source for leather making.

The term tanning (and *tannage* in French) itself has also a root in the word *tan*, and it was originally reserved for leather production with tannins but, since the 19th century with the development of other processes, the word has been applied for all different processes of leather making [11,12].

Vegetable tanning could be performed in different ways mostly depending on the size of animal skins, but since the medieval period three main techniques could be found in Europe: pit, vat and bag tanning [13,14,15,16].

Tanning in pits—large tanks set on the ground—was a slow and static process mostly reserved to the treatment of large animal skins (hides) and to produce heavy and strong leathers for shoe soles, belting or harness. Succinctly, hides were set in horizontal layers inside the pits (or *layaways*) with the ground vegetable material, one layer of hide another layer of crushed barks or leaves. Water or oozes was then added to the pits until all the skins were immersed and left for about 6 to 12 months.

The tanning of small to medium skins was mostly performed in wooden or clay vats where they were put together with the vegetable material, or an aqueous liquor of these materials, and stirred together for a few weeks until the required amount tanning material had been absorbed.

The last process, bag tanning, mostly used in south of Europe, was a faster process since it took only two to three weeks to complete. This method was used to produce light and fancy leathers that were then applied in lavish items such as jewellery and cases coverings, etc. To obtain these exquisite leathers, the skins were folded, sewed like a small bag and filled up with water and with the ground vegetable material inside. Then “bags” were immersed in an identical infusion or liquors used to fill it and shake to speed up the process of tanning. Basils (*badanas*, in Iberian languages), cordovans and morocco are some examples of vegetable tanned leathers traditionally produced by bag tanning technique [17,18,19,20].

With the Industrial Revolution, and the need of faster tanning processes, all vegetable tanned leathers started to be produced in drums which consist of a wooden cylinder rotating around its own axis filled with skins, tanning agents and water.

Leather produced with tannins, or vegetable tanned leather, was one of the most important pre-industrial materials in Western world, very much appreciated and demanded due to its versatility. Its characteristics, ranging from rigid to flexible, depend on the raw materials used, both skins and vegetable tannins, and the tanning techniques. It was a fundamental material for the production a wide range of artefacts such as footwear, garments, book bindings, saddlery, wall-hangings, furniture upholstery, cases coverings, carriages or liquids containers [7,21]. Beyond its utilitarian function, it was also used as support material for artistic and decorative paintings, wall hangings or screen coverings. Different ornamental techniques such as dyeing, painting, gilding, moulding, tooling, embroidering, cutting-out, scorching or sewing, have been often incorporated transforming vegetable tanned leather into a valuable and luxurious material. Gilt leather (Spanish leather, *guadameci*) is one of the finest examples created in Europe: it is a silvered and then “golden” varnished, painted and tooled or moulded vegetable tanned leather, mostly produced between the 16th and 18th centuries [13,22,23].

Therefore, vegetable tanned leather is the most common type of heritage leather found in museums and collections [24,25].

## 2. Vegetable Tanning Materials: The Sources of Tannins

Vegetable tannins are polyphenolic secondary metabolites produced by higher plants. They can precipitate not only proteins, as it was stated before, but also polysaccharides and alkaloids. They are large molecules with a molecular weight comprised from 500 to 30,000 Da. However, not all tannins are useful for tanning, only those with a molecular weight lower than 3000 Da are efficient in leather making since large molecules are unable to penetrate into the skin’s fibre structure and tend to be water insoluble [1,26].

Tannins can be divided in four classes: hydrolysable, condensed, complex and phlorotannins [27,28,29]. Complex tannins have a molecular structure that can be considered a mixture of hydrolysable and condensed, including gallic, ellagic and catechin sub-units. Phlorotannins are a small group of tannins isolated mainly from brown seaweeds. None of the two groups is important for leather tanning.

Hydrolysable tannins consist in a monosaccharide core, usually glucose, esterified with gallic acid, forming the gallotannins, or with hexahydrodiphenoic acid, the precursor of ellagic acid, and gallic acid, forming the ellagitannins. Upon heating in acidic aqueous medium, they hydrolyse to yield gallic and ellagic acid. Thermal decomposition originates pyrogallol which gave the traditional and former name to this class of compounds.

Condensed tannins have a flavonoid origin. They are oligo- or polymeric proanthocyanidins were the phenolic hydroxyls are totally or partially esterified with gallic acid.

Many plants rich in tannins had been used over time. Table 1 lists the most important sources of tannins used in European tanneries until development of mineral tanning since before the 18th century the autochthonous plants were the main raw vegetable tanning materials. However, with the growing need for leather and the increase in trading between Europe and other continents such as Africa, Australia and South America, other exotic sources arrived at Europeans tanneries in large amounts and low cost [15,30,31].

The selection of the appropriate vegetable tanning material was crucial to obtain leathers with specific characteristics [32]. Leathers produced with hydrolysable tannins are light brown, yellow or greenish, and are lightfast. Leathers produced with condensed tannins have a brown to reddish colour, becoming darker if exposed to light. This latter type of leather tends to absorb more easily atmospheric pollutants like sulphur dioxide, promoting the acidic hydrolysis of collagen, leading to its degradation. The so-called red rot is an advanced stage of degradation where cohesion is lost due to the disintegration of collagen fibres. Leather produced with condensed tannins is more prone to this occurrence than the one produced with hydrolysable tannins [9,25].

### 2.1. Barks

Barks from different tree species were, and some still are, very important raw materials in the production of vegetable tanned leathers.

In Europe, oak (*Quercus* spp.) barks were the most used. In the British Isles and other western European countries, the prevailing species were English (pedunculated) oak (*Quercus robur* syn. *Q. pedunculata*) and sessile oak (*Quercus petraea* syn. *Q. sessiliflora*), while in the south predominated Pyrenean oak (*Quercus pyrenaica*) and Portuguese oak (*Quercus faginea*). In the Mediterranean and Atlantic regions of south Europe, the bark of smaller and endemic species belonging to the *Quercus* genus such as cork oak (*Quercus suber*), holm oak (*Quercus rotundifolia* syn. *Q. ilex* subsp. *rotundifolia*) and kermes oak (*Quercus coccifera*) were also used since these species grow spontaneously [12,18,33].

Barks were removed from trees with 12 to 15 years old, between spring and early summer, since it is the time when barks are richer in tannins and, at the same time, easier to separate from the trunk. Barks were dried and then ground into a powder containing 6–17% weight of a mixture of condensed tannins and ellagitannins [1,7].

Early analysis of *Quercus robur* and *Q. petraea* barks found that they contained proanthocyanidins of the procyanidin and prodelphinidin type. Nevertheless only 23% of the water-soluble oak bark tannins consisted of oligomeric proanthocyanidins [34,35].

**Figure 1:**
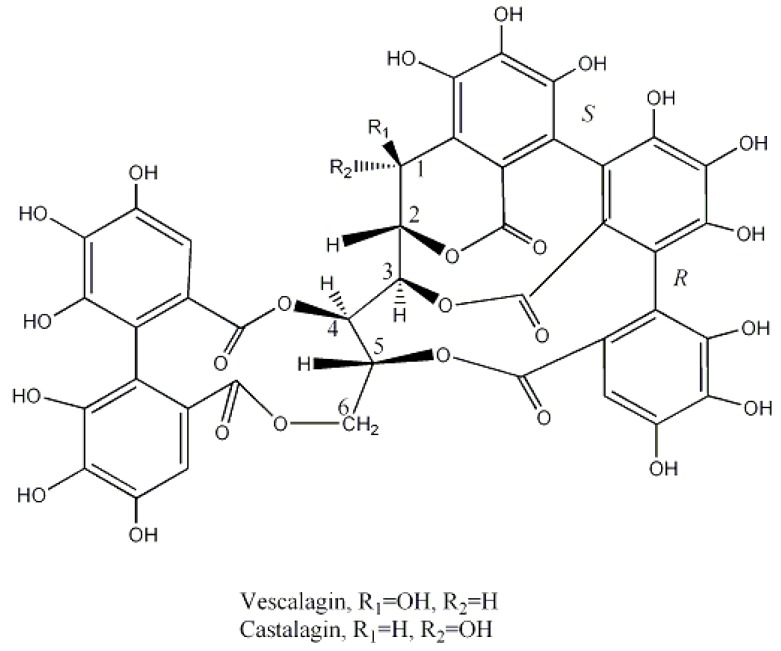
Structure of vescalagin and castalagin.

*Quercus* spp., namely *Q. robur*, such the other species known by the common name of oak, are also a rich source of ellagitannins. The main ellagitannins that account for 40–60% of components of the bark are vescalagin and castalagin. These are complex molecules comprising a linear glucose unit with OH at the 4 and 6 positions esterified with a hexahydrodiphenoic acid (HHDP acid). At the same time, it also exists a C-C coupling between C-1 of glucose and C-2 of a nonahydroxytriphenoyl moyaty. This unit esterifies the remaining OH of glucose at the 3, 4 and 5 positions (Figure 1). Vescalagin and castalagin are epimers at the C-1 position of glucose unit [26,36].

Steriochemistry of the C-C bond between the two galloyl unit, linked to C-3 and C-5 OH of glucose has recently been reinvestigated [37].

**Figure 2:**
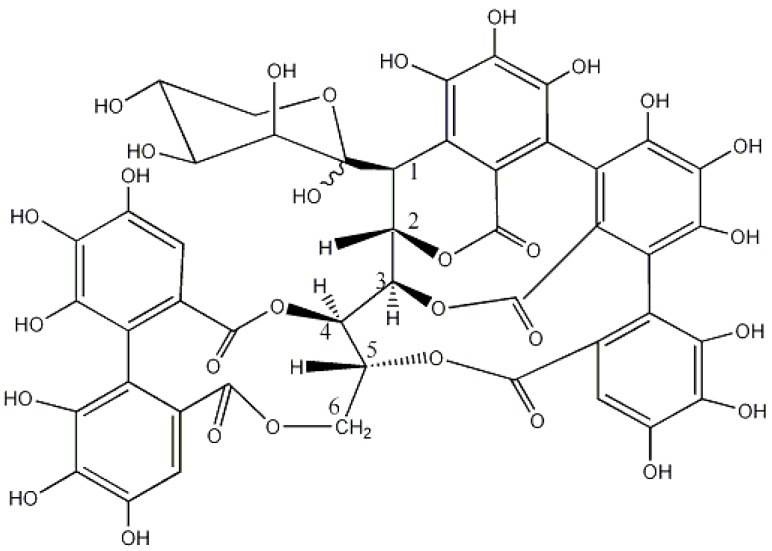
Structure of grandinin.

*Quercus suber*, cork oak, was also used in tanning. The inner part of the bark was removed and used in Portugal, Spain and Corsega. It was also exported to Ireland [20,33,38]. Investigation of cork from Spanish specimen indicates that the main tannins were common to the other *Quercus* species, the ellagitannins grandinin (Figure 2), vescalagin, and castalagin, being grandinin and castalagin the main components [39].

*Quercus coccifera*, kermes oak, was very used in the French Provence where it was known as *garouille*. *Rusque*, the name of the husk of the root, was used to produce shoe sole of high quality [19,20].

In northern Europe and Russia, the main vegetable tanning materials were the barks of local trees like *Betula* spp., birch, *Salix* spp., willow, *Larix* spp., larch, or *Picea* spp., spruce. The use of these barks was restricted to these regions [20,33].

One of the finest examples of vegetable tanned leathers produced in Eastern Europe was Russia leather, which was obtained by tanning the hides or skins with birch or willow barks. It was a highly valued leather, being exported from Moscow to Western Europe from the 17th to the 19th centuries where it was very appreciated for furniture upholstery and lavish coverings [7,18].

The above species biosynthesise condensed tannins although these compounds were found in small percentages [40].

In Scandinavia, barks from *Salix arenaria* and *Salix resseliana* containing 7–11% of condensed tannins were traditionally used to produce leather for gloves [20].

Tannins from the bark of *Picea abies* (Norway spruce) were identified as combinations of (epi)catechin and (epi)gallocatechin units with a polymerization degree of up to 13 units [41].

During the late 18th and 19th century there existed a great demand on tanning vegetable material. To overcome the problem, barks from what was called exotic trees were imported from South America, South Africa, Australia and New Zealand. One of the most imported material was the bark of *Acacia mearnsii,* black wattle, endemic in Australia and New Zealand and cultivated in South Africa. This material is still very used today in Europe by leather industry. Barks were collected from August to October. The outer part was discarded, and the inner part was milled. It could be used as a powder or boiled to produce liquor that was evaporated to dryness and traded for all Europe [20,42]. *Acacia* bark contains 22–48% of condensed tannins. This source of tannins is a very effective tanning agent and is used to produce different types of leathers, with a light red colour when new becoming darker with time.

**Figure 3:**
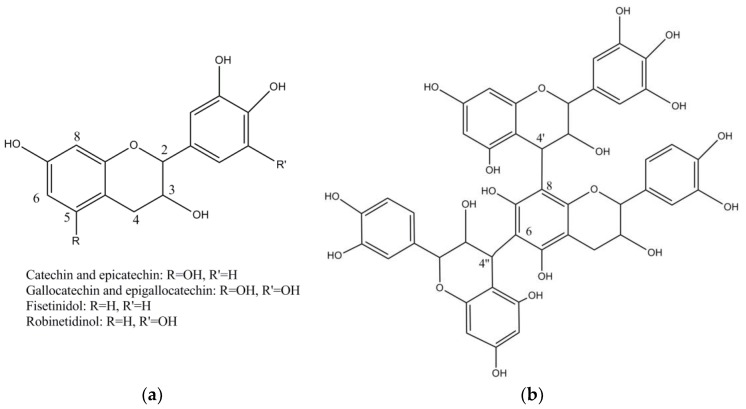
Structure of flavan-3-ol monomers (**a**) and mimosa condensed tannin (**b**).

Wattle tannins consist of about 9% monomers, 42% dimers, 40% trimers, 9% tetramers and 1% pentamers and higher oligomers by mass. The starter unit is either catechin or gallocatechin and the extender units, fisetinidol or robinetinidol (Figure 3a). The second extender unit is always linked to the starter unit to give angular trimers (Figure 3b). The predominance of trimers in the vegetable material is essential for leather tanning since high molecular weight oligomers would not be able to penetrate skin collagen fibres. *A. mearnsii* proanthocyanidin has recently been investigated. The percentage of the different monomers in the tannin of mimosa are around 15% of catechin, 65–70% of robinetidinol and 15–20% of fisetinidol. It can be considered as a mixture of procyanidin or profisetidin and prorobinetidin or prodelphinidin substructures (Table 2) most probably as a mixture of procyanidin and prorobinetidin or prodelphinidin since the proportion of OH/H substitution at position 5 is 6.8:1 and the proportion of OH/H substitution at position 5′ is 3:2 [43,44].

### 2.2. Wood

**Figure 4:**
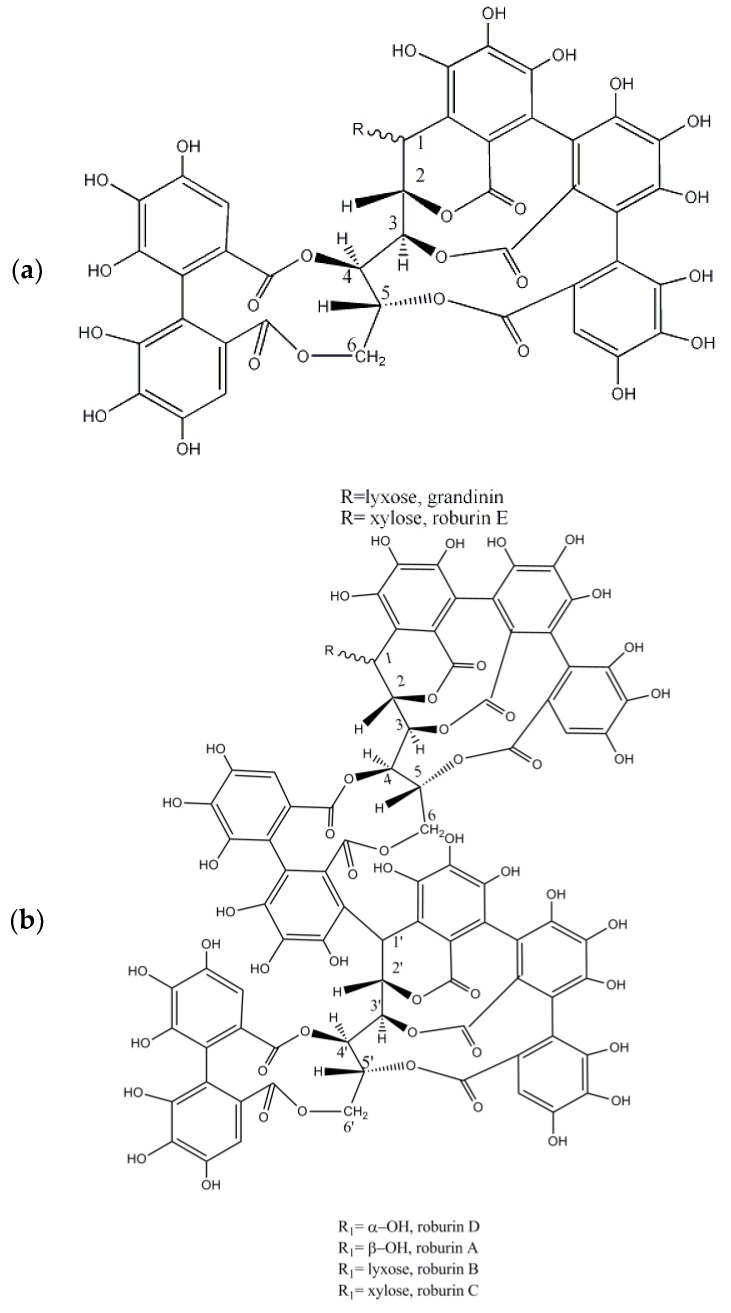
Structure of ellagitanins present in oak wood: (**a**) grandinin and roburin D; (**b**) roburins A, B, C and D.

Wood was mainly used in furniture and buildings, so its use in tanning was scarce until the beginning of the 19th century when an urge need for new tanning materials appeared. Both chestnut and oak wood began to be used as ground or aqueous extracts [45]. The wood of these species, like the barks, are also rich in ellagitannins. Nowadays, chestnut extracts are still used in industry being mainly produced in Italy, Central Europe and Baltic region. Research of chestnut heartwood indicated that has a polyphenolic constitution similar to oak wood. Monomeric vescalagin and castalagin predominate in oak wood representing 40–60% of the ellagitannins. Dimers (roburin A and roburin D, (Figure 4a) and other compounds were other sugar unit like lyxose/xylose derivatives grandinin, roburin B, roburin C, and roburin E (Figure 4a,b), were also identified in oak woods [46]. Some of the previous roburins like roburin A and roburin E were also identified in small amounts in oak bark [39].

**Figure 5:**
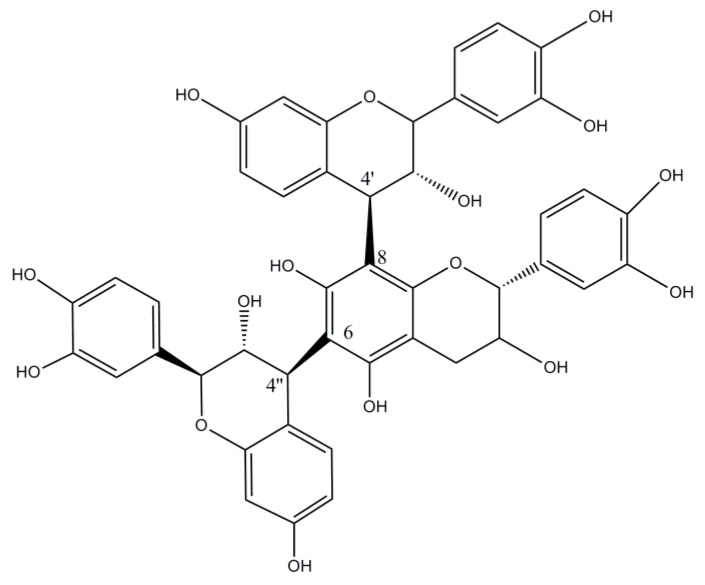
Structure of quebracho tannin.

During the last decades of the 19th century Europe began to import an exotic wood, quebracho, *Schinopsis lorentzii* and *S. balasae*, from south America [18,47]. This tree has hard and reddish wood rich in condensed tannins, about 14–26% weight of the heartwood. Quebracho tannins extracts contains oligomers based on catechin as starter unit linked to one, two, three, etc. *ent*-fisetinidol extender units (Table 2, Figure 5). The trimer is angular with one fisetinidol linked to the C-8 position and the other to the C-6-position of catechin. Analysis of the tannin indicates that is composed by about 33% dimers, 37% trimers, 21% tetramers, 8% pentamers, and 1% heptamers. Compounds with higher degree of polymerisation, if present, exist in low concentration [1,48,49].

### 2.3. Leaves

In Europe, until the 20th century, sumac (*Rhus coriaria*) leaves, together with oak bark, were one of the most important materials used to produce leathers. Sumac has been used since Antiquity and it is even mentioned in Pedanius Dioscorides book “De Materia Medica” written between 50–70 AD. During the Middle Age it was a valuable material used to produce delicate, light colored and great durability leathers. It was also used in the dying of leathers and textiles. In Iberian Peninsula it was used to prepare the famous cordovan, leather prepared with goat skins, and basils. The word sumac refers to several species belonging to the genera *Rhus* of the Anacardean family. It also refers to the powder obtained milling the leaves and small branches of the shrub collected from July to September. Good quality sumac contains 25–35% of gallotannins. *Rhus coriaria* grew spontaneously or cultivated in several regions of the South Europe, namely Portugal, Spain and Sicilia, produced a light yellow to green tanning powder.

During the 17th and 18th centuries there was a great demand for Portuguese sumac produced in the north of the country and it is described in the firsts encyclopedias as having a superior quality. It was exported to France and British Islands to be used by the textile and leather manufactures of Northern Europe. However, during the 20th century Spanish and *Sicilian sumac* surpassed the demand for Portuguese sumac [18,33,50].

In Turkey and in the Oriental Mediterranean another specie of sumac was used, named Turkish or Venezian sumac, *Rhus cotinus* (syn. *Cotinus coggygria*) used in the Mediterranean region but containing less amount of tannins. Both sumacs tanning materials produces pale and soft leathers, contrasting with other tanning agents that originate brown coloured leathers. Pale leathers were much appreciated since they could be dyed in light and bright colours without dark background interference. There are however some drawbacks: sumac infusions ferment and hydrolyse easily and the resulting material has no tanning properties. They were used in vat or bag tanning.

Besides sumac leaves other plants leaves were also used for leather production, such as myrtle, mastic and *rédoul*. The last one is of French provenance, very used in the French regions of Languedoc and Provence and also in Catalonia where was called *redon*. It was also known as French or fake sumac and it was poorer tanning material, with only about 15% of tannins [7,20,51].

**Figure 6:**
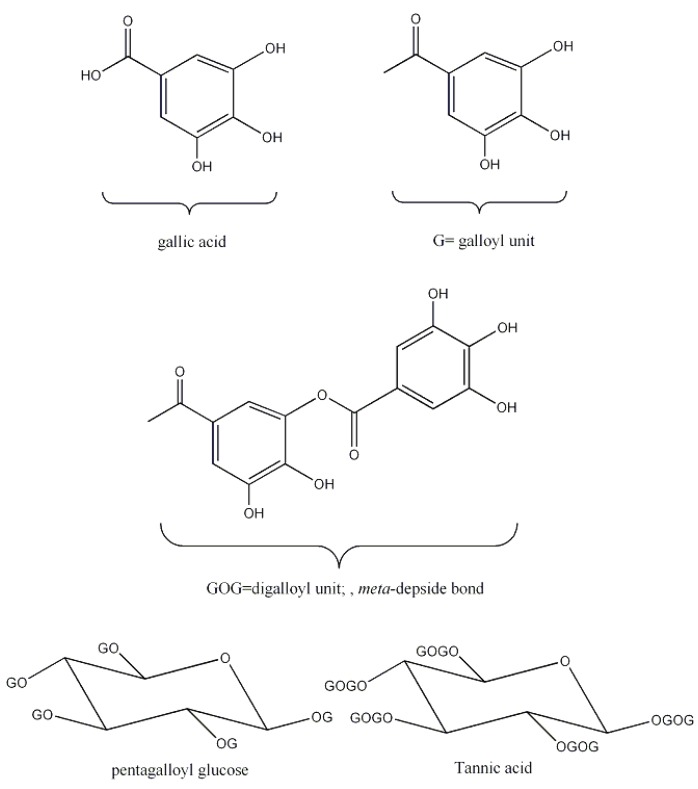
Structure of gallic acid and pentagalloyl glucose and tannic acid

As stated above, sumac is a source of gallotannins. The simplest gallotannin is pentagalloyl glucose, β-1,2,3,4,6-pentagalloyl-*O*-d-glucopyranose (PGG). This compound has the five free hydroxyls of glucose esterified with a galloyl residues. The α anomer is not usual in nature. More galloyl residues can be attached to PGG through a meta- or para depside bond (Figure 6) [26].

Tannic acid is considered the tanning agent in sumac. Commercial tannic acid has a molecular weight of 1701.206 g/mol and a molecular formula of C_76_H_52_O_46_, corresponding to a decagalloyl glucose, sumac leaves contain a mixture of gallotannins, from penta to decagalloyl-glucoside [52,53,54].

In Spain, true sumac was many times forged with mastic (*Pistacia lentiscus*) leaves. However this plant contains condensed tannins and leathers thus obtained were darker and got a reddish colour with light.

Leaves of *Myrtus communis* were widely used for leather production in Italic Peninsula. This plant contains the following ellagitannins: oenothein B, eugeniflorin D2, and tellimagrandins I and II [55].

### 2.4. Fruits

A few fruits were also used as tanning materials, the most important being valonia and divi-divi. Valonia is the acorns of *Quercus aegilops*, a tree also known as Turkish oak, which grows abundantly in Turkey, Greece and adjacent countries. Acorns are usually picked up in August and then dried out from the domes. It has been widely used in the production of leather in Austria, Germany and France and allows producing faster, harder, firmer, heavier and quite impermeable to water leather. Valonia is a source of ellagitannins, whose main constituents are castalagin, vescalagin and pentagalloylglucose (Figure 1 and Figure 6) [56]. This material was often mixed with ground oak bark to produce soles that were considered of excellent quality and durability [18,20].

The divi-divi is a pod of a shrub, *Caesalpinia coriaria*, native to South America. It became to be imported and used in English tanneries in the 19th century. This material, mainly a source of gallotannins containing a small amount of condensed tannins, produces very porous leathers of brown or reddish-brown colour [20,57].

### 2.5. Galls

Galls are pathological excrescences formed in the branches, leaves or domes of plants as a response to the bites of certain insects or other parasites. They are anomalous grow of plant tissues and have different size, shape and composition depending on the plant and the agent that causes them. Galls used in tanning have a spherical (globose) shape, are smooth or have a crown and are characterized by a high content of tannins, 40% to 70% of gallotannins. The most important are the so-called Turkish galls, Aleppo or Levant galls, and are produced by the insect *Cynips gallae tinctoriae* in the branches of the *Quercus infectoria* (syn *Q. lusitanica*) growing mainly in Asia Minor. These galls have a greenish or brownish colour, are rigid, compact, very astringent, bitter to taste and are one of the raw materials used to obtain tannic acid [33].

The so-called white galls, brownish-yellow, lighter, less astringent and with bitter taste, having less tannin content than greenish galls, are another type of galls produced by *Q. infectoria* [19,20]. Since Ancient Greece that both have commercial importance and, in addition to tanneries, were widely used in traditional medicine, dyeing and to make writing ink (iron gallic ink).

Another important gall is known as *knoppern*, or acorn gall, which is caused by the insect *C. quercus-calycis* and found in many Central European *Quercus (Q. robur, Q. pyrenaica)* (Austria, Hungary, Serbia, Slovenia) and Greece [20].

## 3. Detection and Characterization of Tannins in Historic Vegetable Tanned Leathers

Vegetable tanned leathers, i.e., leathers produced with plant materials rich in tannins, are the commonest European cultural heritage leathers.

In heritage conservation, identification of leather making process is important to comprehend leather technology, degradation susceptibility and condition.

The analysis of tannins is a relatively common procedure in historic leather conservation and heritage science studies. Tannins characterisation allows to elucidate aspects on historic leathers composition, technology, and condition, but also to evaluate the suitability of new vegetable tanned leathers for conservation purposes [9,58].

Tannins characterisation in leathers can be based on spot tests, formerly the ferric test and vanillin test and more recently the rhodanine, nitrous acid and the acid butanol tests. These chemical tests are employed for a fast-preliminary material characterisation, especially by leather conservators [58,59,60,61,62,63].

Some studies using chromatographic techniques, namely high-performance liquid chromatography (HPLC) have been also used, but more costly and time consuming and only allows to distinguish the broad classes of condensed versus hydrolysable tannins [64].

### 3.1. Colorimetric Tests

Different colorimetric tests developed in the field of phytochemistry [26] have been adapted for tannins detection in historic and archaeological leathers. These tests, particularly ferrous and vanillin test, have been performed as spot test directly in fibres collected from leathers [61,62,65]. Recently, more specific chemical tests have been adapted to analyse both leather fibres and extracts prepared from those fibres. The analysis of fibres with these tests is done visually. However, the identification of tannins can be difficult to interpret in coloured or aged leathers samples due to the presence of colorants or degradation products of the leather itself. To overcome these limitations, chemical analysis of historic leathers can be performed in leather fibres extracts and analysed spectrophotometrically [66]. 

**Figure 7:**
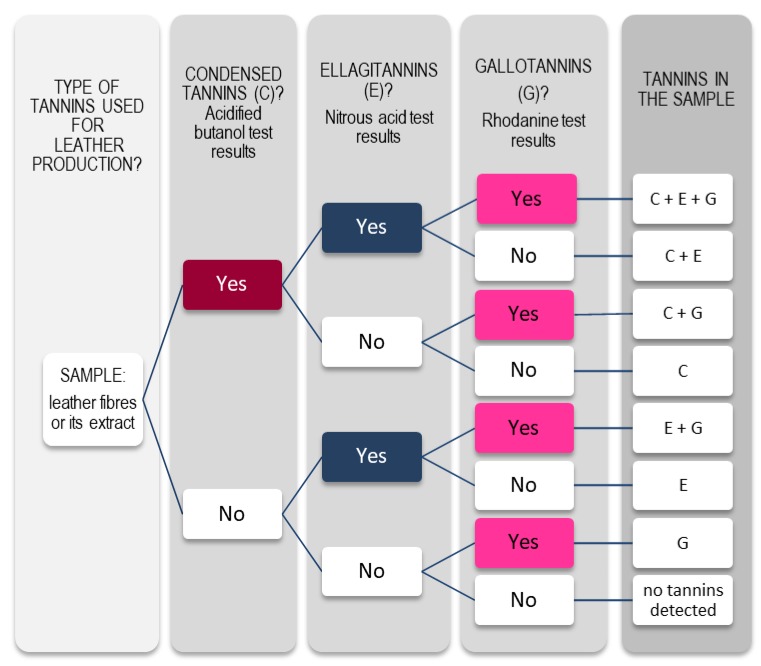
Key-guide for the identification of tannins by colorimetric tests.

Colorimetric tests can be used as a global and fast tool to detect and classify vegetable tannins. When specific tests for the detection of condensed tannins (HCl/butanol test), ellagitannins (nitrous acid test) and gallic acid/gallotannins (rhodanine test) are performed, a combined evaluation of results is possible allowing a more specific detection of the chemical nature of tannins used in the manufacture of the leather (Figure 7) [66].

#### 3.1.1. Ferric Test

This type of test is specific for phenolic compounds and not only tannins. It is based on the principle that phenolics react with iron salts forming a bluish or greenish black (or grey) product [58,64]. Iron salts such as iron sulfate (III) (Fe_2_(SO_4_)_3_) or iron chloride (FeCl_3_) are the commonest reagents used in ferric test.

Some literature refers that this test can be used to distinguish hydrolysable from condensed tannins. Hydrolysable tannins, namely tannic acid, form blue black products while condensed tannins form green black products. This subtle distinction is very difficult to observe in leather fibres, particularly in aged dark leathers or when mixtures of different types of tannins are present and if diverse coloured fibres are to be compared [61,62].

#### 3.1.2. Acidified Vanillin Test

Vanillin test has been widely used to detect condensed tannins. In acid conditions, vanillin reacts specifically with meta-substitued flavanols, not only condensed tannins, to form a red product. Widely distributed flavanols such as catechin and epicatechin, condensed tannins monomers, also react with the disadvantage they give higher colour yield than the condensed tannins. Furthermore, this test is very sensitive to the presence of water that quenchs colour yield. In addition, quebracho proanthocyanidins do not produce an intense colour with this reagent [26].

Reactivity with vanillin cannot be considered sufficient evidence for the presence of condensed tannins [23,67]. Furthermore, vanillin reacts reluctantly with aged leathers, becoming very difficult to undoubtedly identify the presence of condensed tannins [59,61,62,68].

#### 3.1.3. Acidified Butanol Test

Acid butanol test is a depolymerization method specific for condensed tannins detection. With this method it is promoted an oxidative cleavage of interflavanoid bond of proanthocyanidins in hot acidified alcohol solutions to yield correspondent red anthocyanidins with absorbance maxima around 550 nm. The red colour can vary significantly depending on the anthocyanidin formed and on the position of interflavan links (C4 → C6 were found to be more resistant to cleavage than C4 → C8) [23,67].

This test, specific to condensed tannins, is more sensitive than vanillin test and results are unequivocally [62].

#### 3.1.4. Nitrous Acid Test

Ellagitannins react with nitrous acid, obtained by dissolving sodium nitrite in a diluted acid, forming a red or pink colour, which slowly changes to purple or blue [23,67,69].

Nitrous acid test can also be used to detect ellagic acid, an ellagitannins degradation product. With this reagent ellagic acid forms a red chromophore product while other phenolics can form yellow or orange products.

Ellagic acid can be present in aged leather due to acid hydrolysis promoted by moisture and acidic pollutants. The red colour formed in tested historic samples indicates ellagic acid may be present [62].

#### 3.1.5. Rhodanine Test

Rhodanine test is specific for gallic acid detection and was developed by Inoue and Hagerman for gallotannins determination in plant materials. In basic solution, this reagent (2-thio-4-ketothiazolidine) reacts with the vicinal hydroxyl groups of free gallic acid producing a red complex, with a maximum absorbance around 520 nm. This red colour is not formed with galloyl esters of gallotannins, ellagic acid, ellagitannins or condensed tannins. However, this method can be used indirectly to detect gallotannins presence performing the test before and after hydrolysis of tannins [70].

Rhodanine test was adapted to detect free gallic acid in fibres and, indirectly, to estimate gallotannins, without hydrolysing samples, by comparing results with acid butanol and nitrous acid tests. The presence of free gallic acid in historic leathers may be due not only to gallotannins degradation (hydrolysis), but also to ellagitannins, complex tannins and even condensed tannins degradation. Therefore, an unequivocal assumption of a leather tanning with a vegetable source of gallotannins is only possible if ellagitannins and condensed tannins are not detected (Figure 7) [62,66].

### 3.2. Spectroscopic Techniques

#### 3.2.1. UV-Vis

Ultraviolet-visible (UV-Vis) spectroscopy has been used to analyse tannins extracted from European historic leathers, such as bookbinding and upholstery leathers, principally as a complementary technique to other spectroscopic research studies.

It is well established that the different classes of tannins from different vegetable sources present characteristic UV absorption bands. Briefly, and considering hydrolysable tannins, the wavelength of the maximum absorbance (λ_max_) and the respective inflection point (λ_min_) are as follows: while gallotannins show two characteristic absorption maximums, λ_max1_ around 212 nm and λ_max2_ around 275 nm, with distinctive inflection point around 242 nm; ellagitannins present strong absorption near 200 nm and a shoulder around 277 nm. Regarding condensed tannins, they present a strong absorption around 200 nm, an inflection point (λ_min_) between 258–259 nm and λ_max_ between 279–281 nm [71,72].

This distinctive spectral data has been found when analysing leather fibers extracts gathered from leathers of different periods and geographical origins. Studies demonstrate that UV spectra can clearly indicate if gallotannins or condensed tannins are present in extracts obtained from leathers tanned exclusively with one of these types of tannins. UV spectra obtained from samples containing ellagitannins, mixtures of different types of tannins or combined tannages are more difficult to interpret [66].

Other authors [73] present similar values for bookbinding and upholstery leathers.

#### 3.2.2. FTIR

Fourier transform infra-red (FTIR) spectroscopy is a very useful and common analytical technique in heritage science studies and in the last decade different studies have been published describing this technique to analyse tannins in historic leathers [36,66,73,74,75,76,77].

Tannins from different vegetable sources present characteristic absorption bands in mid- infrared region of spectrum. The 1750–700 cm^−1^ region was considered the most informative. All tannins FTIR spectra exhibit four strong bands, two of them at 1615–1606 cm^−1^ and 1452–1446 cm^−1^ assigned to aromatic ring stretch vibrations and the other two at 1211–1196 cm^−1^ and 1043–1030 cm^−1^ assigned to stretch vibrations of C–O bond. Tannins also present another weak band at 1518–1507 cm^−1^ due to skeletal vibration of the aromatic rings.

And it is also demonstrated that the fingerprint region (1800–650 cm^−1^) of hydrolysable tannins presents an absorption pattern distinct from condensed tannins. Hydrolysable tannins presented bands at 1731–1704 and 1325–1317 cm^−1^. The gallotannins sub class present three distinctive bands at 1088–1082, 872–870 and 763–758 cm^−1^.

Vegetable tanning materials classified as condensed tannins, showed three strong bands at 1288–1283 cm^−1^, 1160–1155 cm^−1^ and 1116–1110 cm^−1^ and two other weak bands at 976 and 844–842 cm^−1^. These bands are not found in the spectra of gallo- and ellagitannins. The 1288–1283 cm^−1^ indicates a characteristic feature for the flavonoid-based tannins. This band can be assigned to the ethereal C–O asymmetric stretching vibration arising from the pyran-derived ring structure of this class of tannins [78,79].

The tanning materials in historical leathers can be analysed by FTIR technique after extraction with aqueous acetone, followed by lyophilization. The presence of the four strong bands at 1615–1030 cm^−1^, as referred above, is a strong indication that the material had been tanned with vegetable tannins. Further characterization is possible looking for the marker bands of each class (Table 3). Ellagitannins can only be identified if marker bands for hydrolysable tannins are present and marker bands for condensed and gallotannins are missing. Table 3 presents data obtained with ATR device. If other technique is used like KBr pellet or diffuse reflectance identical or very close values are obtained.

Usually historic leather tanned with sumac like morocco leather are easily identified since the characteristic bands of gallotannins are well defined. However, if leathers were tanned with oak, which contains a mixture of ellagi- and condensed tannins, or if the leather was tanned with more than one type of tannin, FTIR spectra present the characteristic bands of hydrolysable and condensed tannins, do not allowing the differentiation between these two situations.

When spectra display intense bands around 1650 and 1550 cm^−1^ corresponding to the amide I and II bands of collagen, respectively, is an indication that leather had suffered a considerable degradation of the proteinaceous material.

#### 3.2.3. Other Spectroscopic Techniques: Fluorescence Spectroscopy and Solid State ^13^C-NMR

There are few reports than the above mentioned of other spectroscopic techniques used to characterize vegetable tannins in historical leathers.

Emission fluorescence spectra of five tannins, oak, valonea, chestnut, quebracho and mimosa, were recorded between 200 and 800 nm after excitation at 220 nm and 250. Results were used by the authors to confirm the conclusions obtained by FTIR and UV spectroscopy [73].

Solid state ^13^C-NMR was used to distinguish between leather tanned with vegetable material from leather tanned with mineral tanning agents. Special attention was paid to the spectra between about 165–171 ppm. In this region peaks of vegetable tannins are very important while those from collagen are small and scarce. The technique was applied only to new tanned leathers and requires the use of small amount of material, 1–2 mm, which were freeze in liquid nitrogen and then milled to a powder. Besides the distinction between hydrolysable and condensed tannins the authors also to distinguish leather tanned with mimosa from the leather tanned with quebracho [80].

## Figures and Tables

**Table 1:** Post-medieval and traditional vegetable tanning materials used in Europe [18,19,20,33].

Botanical Name	Common Name	Origin and Distribution	Part of the Plant Used	Main Tannins	Geographical Uses	Observations
*Acacia mearnsii*	mimosa, wattle	Australia, cultivated in South Africa since 1864 and South America	barks	condensed	imported since second decade of 19th century, commercial extracts	
*Betula* spp.	birch	northern Europe, Russia	barks	condensed	northern Europe, Russia	used to produce Russia leather
*Caesalpinia coriaria*	divi-divi	Central and South America	pods	hydrolysable: gallotannins	imported since late 18th century	
*Castanea sativa*	chestnut, sweet chestnut	Mediterranean region	wood	hydrolysable: ellagitannins	since 19th century, commercial extracts	used mixed with other vegetable materials to produce firm leather
*Coriaria myrtifolia*	Mediterrenean coriaria (*emborrachacabras*, redoul, roldor, rodor)	southern France and Mediterranean coastal Spain	leaves (redoul)	hydrolysable	southern France and Mediterranean coastal Spain	
*Cotinus coggygria* (syn *Rhus cotinus*)	smoke tree	southern Europe, Mediterranean region	leaves (Venetian or Turkish sumac)	hydrolysable: gallotannins	southern Europe	
*Larix*	larch	northern Europe	bark	condensed	northern Europe	
*Mirtus communis*	myrtle	southern Europe	leaves	hydrolysable: ellagitannins	Italic Peninsula	
*Picea abies*	Norway spruce	Alps, Pyrenees, Germany, Scandinavia	barks	condensed	northern and central Europe	
*Pinus halepensis*	Aleppo pine	coastal areas of the western Mediterranean region	barks	condensed	northern Europe	yields a reddish leather
*Quercus aegilops*	valonea oak, Turkish oak	eastern Mediterranean region	acorn cups	hydrolysable: ellagitannins	Middle Ages in Turkey, Greece, Italy	
*Quercus coccifera*	*garouille*	Mediterranean region	husk of root (*rusque*)	hydrolysable	south of France	
*Quercus infectoria*	Aleppo oak	eastern Mediterranean region	galls (Allepo galls)	hydrolysable: gallotannins	Europe	
*Quercus ilex*	holm oak	central-western part of the Mediterranean	barks	condensed and hydrolysable	Iberian Peninsula	
*Quercus* spp. (*Q. ilex*, *Q. robur*, *Q. petraea*, *Q. pyrenaica*)	oak	Europe	barks	condensed and hydrolysable: ellagitannins	Europe	
			wood	hydrolysable: ellagitannins		
*Quercus suber*	cork oak		inner bark	condensed and hydrolysable: ellagitannins	Iberian Peninsula	
*Rhus coriaria*	sumac	Mediterranean region	leaves (*Sicilian sumac*)	hydrolysable: gallotannins	southern Europe	yields light coloured, soft and supple leathers. Used to produce basil and cordovan leather.
*Salix* spp.	willow	northern Europe, Russia	barks	condensed	northern Europe, Russia	yields a light coloured, yellowish-brown leather that is soft and flexible
*Schinopsis balansae*, *S. lorentzii*	quebracho	south America	wood	condensed	imported and used in Europe since last decades of 19th century	
*Terminalia chebula*	myrabolans	India	fruits	hydrolysable	British Islands	used in mixed tannages for sole leather

**Table 2:** Proanthocyanidins monomers.

Tannins Name				
	Procyanidin	Profisetidin	Prorobinetidin	Prodelphinidin
Chemical structure of the building block	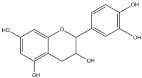	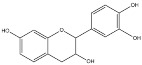	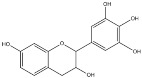	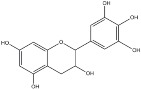
Coupling position	4–8	4–6	4–6	4–8

**Table 3:** Main ATR-FTIR bands of vegetable tanning materials and their assignment [36].

Bands (cm^−1^)	Assignment	Tannin Identification
1731–1704 (m-s)	ν C=O phenolic esters lactones	hydrolysable tannins
	ν C=O phenolic esters	
1615–1606 (m-vs)	ν C=C aromatic ring	present in all classes of tannins
1518–1507 (w-m)	ν C=C skeletal ring	present in all classes of tannins
1452–1446 (m-s)	ν C=C aromatic ring	present in all classes of tannins
1325–1317 (m-s)	ν C-O lactones and O-H deformation	hydrolysable tannins
1288–1282 (ms-vs)	ν C-O pyran ring, flavonoids	condensed tannins
1211–1196 (m-vs)	ν aromatic C-OH	present in all classes of tannins
1162–1155 (s)	ν, asymmetric, C-O-C cyclic ether	condensed tannins
1116–1110 (s-vs)	ν, asymmetric, C-O-C cyclic ether	condensed tannins
1088–1082 (m)	ν, symmetric, C-O-C aryl phenolic ester	gallotannins
1043–1030 (m-vs)	β = C-H deformation	present in all classes of tannins
976 (w)		condensed tannins
844–842 (w)	γ tetrasubsituted aromatic C-H	condensed tannins
872–870 (w)	γ OH and γ tetrasubsituted aromatic C-H	gallotannins
763–758 (w-m)	ν, symmetric skeletal (sugar ring, breathing vibration)	gallotannins

*ν* stretching, *β* in plane, γ out-of plane; vs: very strong, s: strong, m: medium, w: weak.
